# Cognition in multiple sclerosis

**DOI:** 10.1097/WCO.0000000000001481

**Published:** 2026-04-14

**Authors:** Ermelinda De Meo, Emilio Portaccio, Maria Pia Amato

**Affiliations:** a University of Florence, Department of NEUROFARBA, Florence, Italy; b Queen Square Multiple Sclerosis Centre, Department of Neuroinflammation, UCL Queen Square Institute of Neurology, Faculty of Brain Sciences, University College London, London, UK; c IRCCS Fondazione Don Carlo Gnocchi, Florence, Italy

**Keywords:** biomarkers, cognition, multiple sclerosis, phenotypes, treatment

## Abstract

**Purpose of review:**

Cognitive dysfunction in multiple sclerosis (MS) has gained increasing attention over recent decades, reflecting its substantial effects on day-to-day functioning and the limited availability of targeted therapies. This review addresses contemporary advances in the role of cognition to detect disease progression, examines biological and MRI correlates of cognitive dysfunction, and summarizes the evidence for treatment effects.

**Recent findings:**

Cognitive changes can capture both acute relapse–related drops (including isolated cognitive relapses) and gradual decline consistent with progression independent of relapse activity (PIRA). Among fluid markers, serum neurofilament light chains relates to cognition mostly in relapsing disease, whereas glial fibrillary acidic protein seems to track global progression more than cognitive changes. Cerebrospinal fluid (CSF) candidate markers (CHI3L1, parvalbumin) and synaptic proteins (SNAP-25, neurogranin, β-synuclein) may help identifying patients at higher risk of cognitive decline. MRI demonstrates that grey-matter pathology best explains long-term cognitive trajectories while newer readouts (radiomics, quantitative susceptibility mapping of deep-grey nuclei, structural–functional disconnection and multiplex network indices, and choroid-plexus/glymphatic measures) add mechanistic and prognostic specificity beyond lesion burden and bulk atrophy. Data-driven cognitive phenotyping yields reproducible, biologically anchored profiles that outperform dichotomous impaired/preserved labels. Therapeutically, higher-efficacy disease-modifying therapies show the clearest association with preserved processing speed; cognitive rehabilitation, augmented in some settings by transcranial direct-current stimulation, produces additional gains.

**Summary:**

Routine assessment and monitoring of cognitive functions should be embedded in MS care to detect relapse-related changes and progressive decline. Identifying fluid and MRI biomarkers of cognitive dysfunction may help individuate novel targets and specific treatments.

## INTRODUCTION

Between 34% and 70% of adults with multiple sclerosis (MS) experience cognitive impairment [1,2], which is reported across all subtypes – including clinically isolated syndrome (CIS), early relapsing–remitting (RR) MS, and even radiologically isolated syndrome (RIS) – suggesting that deficits may predate clinical onset [3]. However, progressive MS has the highest prevalence of cognitive deficits [1,4].

Cognitive impairment in MS is heterogeneous, spanning attention, memory, executive and visuospatial functions; however, information-processing speed is the most commonly affected [5]. Accordingly, the Symbol Digit Modalities Test (SDMT), which primarily indexes processing speed, has become the most widely used clinical tool for screening and monitoring cognition in MS [6]. However, alongside this screening tool, several multi-domain batteries [e.g. Brief International Cognitive Assessment for Multiple Sclerosis (BICAMS) [7], Brief Repeatable Battery of Neuropsychological Tests (Rao's BRB) [8], Minimal Assessment of Cognitive Function in Multiple Sclerosis (MACFIMS)] [9] allowing a more detailed characterization of cognitive functioning. 

**Box 1:**
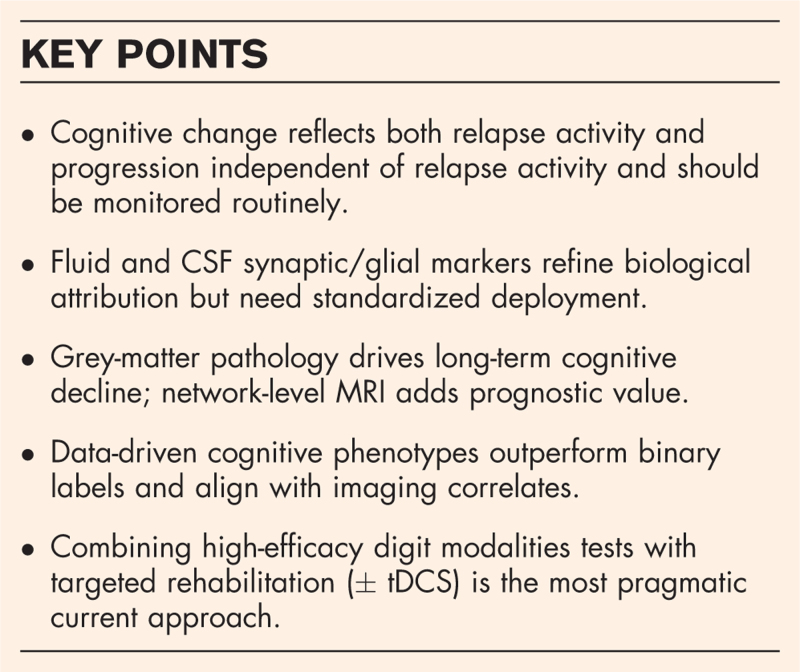
no caption available

## THE ROLE OF COGNITIVE FUNCTIONING IN THE DEFINITION OF DISEASE ACTIVITY AND PROGRESSION

A key requirement for comprehensive clinical evaluation in MS is the routine inclusion of cognitive assessment to detect acute changes attributable to relapses and progressive decline signaling disease progression. In the acute setting, several studies have reported reductions in SDMT scores during relapses that coincide with gadolinium-enhancing lesions on MRI, with recovery often incomplete [10–13]. Emerging evidence also supports the concept of an ‘isolated cognitive relapse’, in which cognitive change is the sole indicator of disease activity, occurring without accompanying sensorimotor symptoms [11,14].

In contrast to the episodic nature of cognitive relapses, progressive cognitive decline in MS is characterized by a slow and steady deterioration of cognitive function over time. This gradual worsening often goes unrecognized until it significantly affects daily activities [15]. Considering this aspect, it is evident that progressive cognitive decline may be as relevant as the expanded disability status scales (EDSS) worsening in detecting the progression independent from relapse activity (PIRA).

Fuchs and colleagues, as firsts, introduced the concept of cognitive PIRA, defining cognitive decline as PIRA when no clinical relapse was recorded between assessments or within nine months of the observed decline [16^▪^]. Using this definition, they found that, in RRMS, most cognitive decline reflected cognitive PIRA and, in 68% of cases, occurred independently of EDSS worsening [16^▪^]. Consistent with this, combining EDSS with the SDMT resulted more sensitive for detecting disability progression in RRMS, particularly in those patients with higher baseline disability (EDSS > 4) [17]. Interestingly, also in this case cognitive decline was independent from clinical disability progression, while being associated with worse quality of life [17]. Given the importance of fatigue for quality of life, a subsequent study broadened the definition of PIRA to include worsening fatigue alongside cognitive decline; once again, changes in fatigue and cognition did not correlate with EDSS worsening, yet this broader ‘progression’ criterion identified more patients with PIRA [18].

## BIOLOGICAL SUBSTRATES FOR COGNITIVE DECLINE IN MULTIPLE SCLEROSIS

Over the last years, serum biomarkers as neurofilament light chain (sNfL) and glial fibrillary acid protein (GFAP) have proven their utility as biomarker for disease activity and progression [19^▪▪^] while their role in determining cognitive functioning is still debated. Several studies reported correlation between sNfL levels and cognitive impairment, as assessed by SDMT performance, Paced Auditory Serial Addition Test (PASAT), California Verbal Learning Test-II (CVLT-II), or Montreal Cognitive Assessment (MoCA) scores [20,21]. Most of these studies only included relapsing MS, with only limited evidence reporting the association of sNfL with cognition in patients with progressive MS [22]. Conversely, although GFAP is even more strongly associated with disability progression than sNfL, no significant associations have been reported with concurrent or future cognitive decline [23,24].

Inflammatory and oxidative markers added nuance: higher tumor necrosis factor alpha (TNF-α) was associated with worse cognition [25–27]. Single studies have also reported associations between lower native and total thiol levels and cognitive impairment [28] as well as between elevated interleukin (IL)-17A [27] and cognitive impairment, and correlations between IL-6 and C-reactive protein with cognitive scores [29]. By contrast, IL-10, glutathione peroxidase, reduced glutathione, catalase activity, ischemia-modified albumin, IL-8, IL-18, and IL-2 showed no association with cognition [25]. Evidence for interferon-γ, total antioxidant capacity, and malondialdehyde remains inconclusive [30,31].

Beyond inflammation, higher serum brain-derived neurotrophic factor (BDNF) levels –interpreted as indexing greater neuroplasticity – were linked to better cognitive performance [32], and higher leptin was associated with worse baseline SDMT with a trend towards subsequent worsening [33].

Extending to cerebrospinal fluid (CSF), and alongside intrathecal B-cell activation indexed by the κ free light-chain ratio [34,35], several additional markers related to cognition. Echoing serum findings, higher CSF NfL concentrations resulted significantly associated with poorer cognitive performance [36], with similar associations reported also for CSF BDNF [32]. More recently, elevated CSF chitinase-3-like protein 1 (CHI3L1/YKL-40) – a chitinase-like glycoprotein secreted predominantly by activated astrocytes and microglia – has been linked to worse cognition, particularly in progressive MS.[37] In addition, CSF parvalbumin, a calcium-binding protein expressed by fast-spiking GABAergic interneurons, was found to be related to the severity of cognitive impairment at diagnosis [38] and able to predict four-year cognitive decline [39^▪^].

While the biomarkers we mentioned so far, mostly relate to neurodegeneration or immune-pathways, growing evidence suggests that synaptic damage and dysfunction may play a key role in the pathogenesis of cognitive decline in MS [40] – much as in neurodegenerative diseases [41,42] – given the central importance of synaptic integrity to the brain networks supporting cognition [43]. In line with this, a recent study reported decreased CSF concentrations of SNAP-25, neurogranin and β-synuclein in MS patients with cognitive impairment [44^▪▪^].

## NOVEL MRI BIOMARKERS FOR COGNITIVE IMPAIRMENT IN MS

In addition to established MRI markers – grey-matter atrophy, lesion burden, and white-matter damage [5] – recent studies have explored newer biomarkers, assessing not only their cross-sectional associations with cognition but also their ability to predict long-term performance. Together, these advances have sharpened the link between structural and network-level pathology and cognitive outcomes in multiple sclerosis, moving beyond lesion counts to mechanisms that better explain domain-specific deficits and inter-individual heterogeneity.

One emerging approach applied radiomics to routine clinical MRI to identify substrates of SDMT performance. Using this method, deep grey matter – particularly the thalamus – showed the strongest associations with SDMT. The derived radiomic features quantified voxel-intensity distributions and spatial relationships (texture, heterogeneity, and regularity) within regions of interest, providing indirect signatures of neurodegeneration and demyelination beyond simple volumetry [45^▪▪^]. Still moving beyond volumetry, a recent study linked increased deep-grey matter susceptibility – interpreted as iron-related tissue injury – to cognitive dysfunction, reinforcing a mechanistic bridge between iron-laden neurodegeneration in thalamus/putamen and cognitive performance [46^▪^]. Finally moving from structure to function the impaired thalamic ability to drive brain state dynamics, as described by using resting-state functional connectivity, was proven to significantly contribute to cognitive impairment in MS [47^▪^].

During the last year brain connectivity has gained increasing interest as substrate of cognitive functioning. A large MAGNIMS analysis integrating structural, diffusion and resting-state fMRI showed that disruption of the brain's multiplex core–periphery organization is greater in patients MS and relates to disability and SDMT impairment, adding explanatory value beyond conventional measures [48^▪^]. A joint-independent component analysis (ICA) fusion of lesion maps, white matter integrity, grey-matter volume and functional connectivity identified co-fluctuating structural–functional patterns (notably involving thalamic radiations, corpus callosum and sensorimotor/thalamic regions) that distinguished MS from controls and explained cognitive dysfunction better than traditional MRI [49]. In another study, joint-ICA combining grey- and white-matter features was associated with all cognitive domains and outperformed single-tissue ICA for executive function and visual memory, underscoring the opportunity to individuate domain-specific mechanistic substrates by applying this technique [50^▪^]. Finally, using only conventional MRI, subject-level structural disconnection and morphometric similarity networks were sensitive to MS-related damage, explained physical and cognitive disability (including SDMT) and – critically – predicted long-term confirmed disability progression independently of lesion burden and atrophy, positioning network-based metrics as practical candidates for prognosis and monitoring [51^▪^].

When examining long-term predictors of cognitive function, grey-matter pathology remains the dominant factor. Longitudinal cortical pathology has emerged as a domain-specific driver of decline: in a 10-year cohort, higher cortical lesion load and the development of new cortical lesions were associated with steeper worsening in attention, verbal memory, and executive function, independently of global atrophy [52]. This indicates that cortical demyelination shapes cognitive trajectories in a functionally selective manner. Consistent with this, a separate 20-year follow-up study showed that greater grey-matter atrophy in eloquent regions – including the precuneus, insula, parahippocampal gyrus, and cingulate cortex – was linked to more severe cognitive impairment two decades later [53].

Finally, among novel MRI biomarkers likely to be explored in greater depth over the coming years, the choroid plexus – and, more broadly, the glymphatic system – deserves particular attention. Associations between larger choroid plexus volume and cognitive performance have already been reported, and measures of choroid plexus microstructural integrity have predicted two-year cognitive outcomes [54^▪^]. Collectively, these findings suggest that choroid plexus pathology may mediate glymphatic dysfunction, which is itself linked to cognitive impairment in MS [55^▪^]. The therapeutic implication is compelling: strategies that enhance glymphatic function could offer a tractable target for treating cognitive dysfunction in MS.

## PHENOTYPING COGNITION: WHERE WE ARE?

Over the past decade, empirical work has shifted from binary “impaired/preserved” labelling towards delineating reproducible cognitive phenotypes in MS, yielding a more granular picture of deficit patterns and their biological correlates. Using a conservative, rule-based approach, Hancock and colleagues applied Jak/Bondi criteria across five domains – attention/processing speed, executive function, language, visuospatial ability and memory – and, at commonly used cut-offs (1.0 and 1.5 SD below normative means), grouped patients by the number of impaired domain scores (0–1 “intact”, 2–3 “single-domain”, 4–5 “bi-domain”, ≥6 “multidomain”); attention/processing speed emerged as the most frequently affected domain [56]. Extending beyond purely cognitive metrics, Podda *et al.* employed latent class analysis incorporating mood measures (anxiety and depression) and identified four clinically distinct profiles: isolated memory difficulties; minor memory–language deficits with mood disturbance; moderate impairments spanning memory, language and attention; and a severe, widespread pattern encompassing memory, language, attention, information processing and executive control [57].

Using a fully data-driven framework, we derived five phenotypes – preserved cognition; mild verbal memory/semantic fluency; mild multidomain; severe executive–attention; and severe multidomain – and showed convergent validity through distinctive MRI signatures for each class, supporting the biological plausibility of phenotype boundaries [58^▪^]. In an independent data-driven study, Van Dam and colleagues likewise identified separable phenotypes, notably one anchored by visuospatial memory deficits [59], while a further application of the same methodology to BICAMS combined with the Beck Depression Inventory-II and Modified Fatigue Impact Scale revealed a subgroup with isolated neurobehavioral impairment, underscoring the salience of mood and fatigue as integral components of the cognitive profile in MS [60].

Across studies, group-level cognitive differences between MS cohorts and healthy controls were consistent yet often modest, frequently falling short of traditional 1.0–1.5 SD thresholds [61]; as a result, threshold-based classifications tended to under-detect subtle but clinically meaningful change and to amalgamate heterogeneous patients within the “impaired” category [62]. Concordantly, the SDMT – while the most commonly flagged measure under dichotomous rules – proved multifactorial, reflecting contributions from processing speed, working memory, visuoperceptual processing, language, sensory–motor functions and executive control; in data-driven solutions, SDMT impairment clustered within more severe phenotypes rather than acting as a singular marker of isolated processing-speed loss. Taken together, these results demonstrate that cognitive impairment in MS is structured, with recurring, biologically anchored phenotypes that better capture the breadth and patterning of deficits than binary labels. They also show that integrating affective and fatigue measures refines subgroup detection and that multivariate, data-driven models offer superior construct validity relative to test-by-test thresholds. Although rule-based systems remain simple to apply to individuals, the cumulative evidence indicates that phenotype-level classification, supported by imaging correlates, yields a more faithful account of cognitive heterogeneity and provides a stronger platform for targeted rehabilitation and future mechanism-based therapies.

## TREATMENT FOR COGNITIVE DECLINE

One of the main findings related to the treatment opportunity for cognitive dysfunction comes from the post-hoc analysis of OPERA I/II reporting greater SDMT gains over 96 weeks with ocrelizumab than interferon β-1a, reinforcing treatment effects on processing speed beyond relapse control; complementary appraisals suggest natalizumab may likewise preserve cognition alongside reduced lesion accrual and atrophy [63^▪▪^]. Cognitive rehabilitation produced measurable but often transient benefits: a multicenter randomized trial of the Guttmann NeuroPersonalTrainer improved verbal memory and increased MRI-derived network connectivity [64], while the CogEx program in progressive MS demonstrated regional grey-matter and network changes with modest transfer to primary processing-speed outcomes [65]. Non-invasive neuromodulation emerged as a valuable adjunct: systematic reviews indicate that transcranial direct current stimulation (tDCS) paired with cognitive rehabilitation yields small-to-moderate improvements (notably processing speed/working memory), with growing feasibility for home-based delivery [66]. Overall while no therapy is licensed specifically for cognition, combining high-efficacy disease modifying treatment with cognitive rehabilitation and neuromodulation could be the most valuable approach.

## CONCLUSION

Taken together, cognitive function should be treated as a core dimension of MS activity and progression. Routine, serial assessment – at minimum the SDMT, complemented when feasible by brief batteries – detects both acute relapse-related drops, including occasional isolated cognitive relapses, and the slow declines that characterize PIRA. Interpretation should be anchored in convergent biology. Among fluids, sNfL relates to cognition primarily in relapsing disease while emerging CSF markers (e.g., CHI3L1, parvalbumin) and synaptic proteins (SNAP-25, neurogranin, β-synuclein) strengthen the link to neuronal network integrity. On MRI, grey-matter pathology – cortical lesions and regional atrophy, especially thalamus and limbic hubs – remains the most informative long-term predictor, while newer readouts (QSM iron in deep nuclei, thalamic radiomics, structural–functional network indices, and possibly choroid-plexus/glymphatic metrics) add mechanistic and prognostic specificity. Clinically, higher-efficacy DMTs show the most convincing signal for preserving processing speed, but no agent is licensed for cognition; structured cognitive rehabilitation, potentially augmented by tDCS, offers additional albeit often transient gains. Moving forward, consensus on test panels, thresholds and practice-effect handling, phenotype-aware multimodal monitoring, and longitudinal validation against patient-centered outcomes will be essential to embed cognitive phenotyping in routine care and to target interventions to those most likely to benefit.

## Acknowledgements

*None.*

### Financial support and sponsorship

*None.*

### Conflicts of interest

*There are no conflicts of interest.*

## REFERENCES AND RECOMMENDED READING

Papers of particular interest, published within the annual period of review, have been highlighted as:

▪ of special interest
▪▪ of outstanding interest
